# The Use of Food Images and Crowdsourcing to Capture Real-time Eating Behaviors: Acceptability and Usability Study

**DOI:** 10.2196/27512

**Published:** 2021-12-02

**Authors:** Katharine Harrington, Shannon N Zenk, Linda Van Horn, Lauren Giurini, Nithya Mahakala, Kiarri N Kershaw

**Affiliations:** 1 Northwestern University Feinberg School of Medicine Chicago, IL United States; 2 National Institute of Nursing Research Bethesda, MD United States; 3 National Institute on Minority Health and Health Disparities Bethesda, MD United States; 4 Lake Forest College Lake Forest, IL United States

**Keywords:** ecological momentary assessment, eating behaviors, crowdsourcing, food consumption images, food image processing, mobile phone

## Abstract

**Background:**

As poor diet quality is a significant risk factor for multiple noncommunicable diseases prevalent in the United States, it is important that methods be developed to accurately capture eating behavior data. There is growing interest in the use of ecological momentary assessments to collect data on health behaviors and their predictors on a micro timescale (at different points within or across days); however, documenting eating behaviors remains a challenge.

**Objective:**

This pilot study (N=48) aims to examine the feasibility—usability and acceptability—of using smartphone-captured and crowdsource-labeled images to document eating behaviors in real time.

**Methods:**

Participants completed the Block Fat/Sugar/Fruit/Vegetable Screener to provide a measure of their typical eating behavior, then took pictures of their meals and snacks and answered brief survey questions for 7 consecutive days using a commercially available smartphone app. Participant acceptability was determined through a questionnaire regarding their experiences administered at the end of the study. The images of meals and snacks were uploaded to Amazon Mechanical Turk (MTurk), a crowdsourcing distributed human intelligence platform, where 2 Workers assigned a count of food categories to the images (fruits, vegetables, salty snacks, and sweet snacks). The agreement among MTurk Workers was assessed, and weekly food counts were calculated and compared with the Screener responses.

**Results:**

Participants reported little difficulty in uploading photographs and remembered to take photographs most of the time. Crowdsource-labeled images (n=1014) showed moderate agreement between the MTurk Worker responses for vegetables (688/1014, 67.85%) and high agreement for all other food categories (871/1014, 85.89% for fruits; 847/1014, 83.53% for salty snacks, and 833/1014, 81.15% for sweet snacks). There were no significant differences in weekly food consumption between the food images and the Block Screener, suggesting that this approach may measure typical eating behaviors as accurately as traditional methods, with lesser burden on participants.

**Conclusions:**

Our approach offers a potentially time-efficient and cost-effective strategy for capturing eating events in real time.

## Introduction

### Background

Poor diet quality is a significant risk factor for multiple noncommunicable diseases, including diabetes, certain cancers, and cardiovascular disease [1-3]; however, effective strategies for promoting healthful dietary behavior changes remain elusive. Data reported by the American Heart Association show that <2% of US adults consume an ideal diet [4], a finding further supported by similar data indicating suboptimal intake in most countries [5]. Changing eating behaviors is challenging, partly because of the multifactorial influences on eating decisions. These range from individual and family-level beliefs, preferences, and constraints to larger social, physical, environmental, and temporal and situational cues [6-9]. Such complexity surrounding eating decisions suggests the importance of documenting not only what people eat but also the social and contextual factors potentially influencing their choices.

A growing number of studies have used ecological momentary assessments (EMAs) to simultaneously capture information on what people eat and the role that various social and contextual factors play in influencing those decisions on a micro timescale (across eating events over 1 or several days) [10]. EMAs involve repeatedly sampling participants’ behaviors and experiences in real time within their natural environments [11]. This typically involves administering surveys several times throughout the day using SMS text messaging or a smartphone app. EMA has been used in several studies to evaluate the predictors of intraindividual changes in eating behaviors throughout the day or across days [12-14]. This timescale and the widespread use of smartphones simplify the evaluation across a wide range of predictors, including stress, social and physical environments, and time of day.

To confidently determine the predictors of eating behaviors, we need to accurately capture eating events. Measuring eating behaviors on a micro timescale makes some of the more traditional self-reported dietary measures less practical or useful [15,16]. For example, a 24-hour dietary recall is difficult and burdensome for participants to document through SMS text messaging or a smartphone app. In addition, this format of data collection would require participants to recall their emotions at the time of the meals or provide further details regarding their environment during each meal, which could lead to measurement error and recall bias. Image-based food data collection methods have been developed and evaluated for measuring energy intake; however, they often require participants to use a fiducial marker when taking the images, followed by time-intensive analyses by a dietitian [17,18]. This approach is useful when quantifying total energy intake or when nutrients are of central importance but less so for measuring the predictors of fluctuations in eating behaviors throughout the day or from day to day (eg, snacking or unhealthy eating events).

Most EMA studies seeking to measure the predictors of eating behavior on a micro timescale require that participants record their eating events through diaries or journal entries [19,20] or through the completion of checklists having a variety of different food types [21-24]. This is problematic, as such lists are finite and may fail to fully capture the relevant food options, especially on a smartphone screen. In addition, the act of checking a box to confirm certain eating decisions may influence and alter behaviors [25]. Thus, an alternative approach that is more comprehensive but less overt is warranted.

### Objective

This study assesses the feasibility—the ease of use and participant acceptability—of coupling participant-captured images with crowdsourcing to document eating events in real time. Collecting images facilitates and enhances the self-reported measures of food consumption. Photographing food is now commonplace and socially acceptable, thereby offering a practical strategy for obtaining comprehensive assessments of eating behaviors while lessening the burden on participants. Furthermore, the use of crowdsourcing to classify and quantify food items is a time- and cost-effective, scalable approach with proven accuracy in other biomedical applications [26]. Crowdsourcing minimizes participant burden by eliminating the need to label food images themselves or fill out dietary recall journals and surveys. Through this elimination, crowdsourcing also limits participant reflection on their eating decisions, which could alter their behaviors during the study time frame. Implemented together, participant-captured images and crowdsourcing of image labels can provide a feasible alternative to current food intake EMA methods.

## Methods

### Study Population

We recruited a convenience sample of 48 former participants of the Chicago Healthy Eating Environments and Resources Study (CHEERS) to participate in this pilot study. CHEERS was a cross-sectional study of 228 nonpregnant women aged 18-44 years and living in 4 racially, ethnically, and socioeconomically diverse neighborhoods in Chicago, Illinois, who could understand English or Spanish [27]. The women were recruited via flyers posted in neighborhood stores, presentations to parent organizations at schools, and mails sent using commercially available address lists. This study focused on recruiting women because of the persistent racial and ethnic obesity disparities that exist among women and as women are typically responsible for food preparation and purchase in their families. Women in this age group were selected, as these years are a critical period for an increase in weight because of a range of factors, including postpregnancy weight retention and declining muscle mass and muscle strength [28]. Data for the original study were collected between 2016 and 2017, whereas data for this pilot study were collected between 2018 and 2019. This study was approved by the Northwestern University institutional review board (STU00203035), and all participants gave informed consent.

### Study Procedure

The CHEERS EMA pilot study comprised a 1-day initial visit, a 7-day EMA study period, and a 1-day final visit. Participants were incentivized to participate with cash rewards: US $20 for the first visit, US $9 per day of valid data collection (up to US $63), and US $30 for the final visit. During the initial visit, participants were asked to complete several questionnaires adopted from other studies or created specifically for CHEERS. Participants also completed the validated Block Fat/Sugar/Fruit/Vegetable Screener, which provides estimates of saturated fat, trans fat, total sugars, *added sugars* (in sweetened cereals, soft drinks, and sweets), fruit and fruit juice, vegetable intake, glycemic load, and glycemic index [29]. In this study, the Block Fat/Sugar/Fruit/Vegetable Screener was used to estimate the usual weekly intake of fruits, vegetables, sweet snacks, and salty snacks.

During the 7-day EMA study period, participants documented all their meals and snacks by capturing images via smartphones using 1 of 2 apps: LifeData (LifeData, LLC) or Mobile EMA (ilumivu, Inc). During the initial visit, study team members installed the apps either on participants’ personal smartphones or on a study-provided smartphone, and participants were trained to use the apps. Study data were stored within the app and then uploaded to the server when connected to Wi-Fi during the study period or when the phone was returned at the end of data collection; therefore, Wi-Fi was not required for data collection.

A combination of event- and signal-contingent EMA surveys were used. For the event-contingent surveys, participants answered 3 short questions at the time each food item image was uploaded. Participants were asked to upload a picture of their meal or snack at the time of each eating episode, and they received an SMS text message every morning reminding them to complete the meal or snack event-contingent surveys. Participants indicated whether the food item was a meal or snack. Trained staff contacted the participants on day 2 of the 7-day data collection period to answer questions and encourage adherence. Signal-contingent surveys were randomly sent throughout the day to assess the role of stress and affect on eating behaviors. Participants were asked to complete 4 surveys per day. They received prompts between 8 AM and 8 PM, with at least 2 hours between prompts. Participants’ phones were set to allow push notifications to alert them as the prompts came through. Each survey was available for 10 minutes to more accurately capture stress and affect in real time. If the meal or snack was not photographed, participants were requested to write down what they ate and upload an image of that description. During the study, participants also wore heart rate monitors and accelerometers to assess their physical activity and stress levels. Participants were rewarded US $4 for each day with valid heart rate and accelerometer data and US $5 for each day with at least 3 signal-contingent and 2 event-contingent EMA surveys.

### Usual Food Intake

Participants completed the Block Fat/Sugar/Fruit/Vegetable Screener [29] to assess their usual intake of foods relevant to the study. The Block Fat/Sugar/Fruit/Vegetable Screener (Screener) is a food frequency questionnaire that has been validated for providing estimates of saturated fat, trans fat, total sugar, fruits, and vegetables. Food frequencies were determined through participant responses to “How many days per week?” for the relevant survey questions (Textbox 1). Participants were asked to select either *none or less than 1*, *1 day*, *2 days*, *3-4 days*, *5-6 days*, or *every day*. Responses were coded as 0, 1, 2, 3.5, 5.5, and 7 and summed by food category. This measure was used to reflect the number of times per week an individual usually consumed that particular food group, and it was compared with the number of times calculated from the images they submitted. As with Amazon Mechanical Turk (MTurk)–processed images, the Screener aimed to capture the number of times each type of food was eaten, not the serving size.

**Questions taken from the Block Fat/Sugar/Fruit/Vegetable Screener at previsit to determine the self-reported intake of fruit, vegetables, salty snacks, and sweet snacks.**

**Fruit**

- Any kind of fruit, fresh or canned (not counting juice)

**Vegetables**

- Green salad and vegetables you put in green salad
- Potatoes, not fried, such as baked or mashed
- Vegetable soup or stew with vegetables
- All other vegetables you eat as a side dish or in any kind of dish, not counting salad or potatoes

**Salty snacks**

- French fries, home fries, and hash browns
- Snack chips such as potato chips, tortilla, corn chips, Fritos, Doritos, or popcorn (not pretzels)
- Crackers such Ritz, soda crackers, Cheez-Its, or any other snack cracker

**Sweet snacks**

- Ice cream and ice cream bars
- Donuts
- Cake, cookies, or snack cakes such as cupcakes, Twinkies, or any other pastry
- Pie, including fast food pies or snack pies
- Chocolate candy such as chocolate bars, M&Ms, Mars Bars, and Reeses
- Any other candy (not chocolate) such as hard candy, Lifesavers, Skittles, and Starburst

### Acceptability of Using EMA to Capture Meals and Snacks and Data Quality

After the EMA study period, participants were asked to rate their experiences with the process of taking pictures of their meals and snacks. Specifically, on 5-point Likert scales, participants were asked how often they remembered to take and upload pictures of their meals and snacks (response options included none of the time [value=1], some of the time, half of the time, most of the time, or all of the time [value=5]), how much taking the pictures changed their eating behaviors (not at all=1, slightly, somewhat, moderately, or substantially=5), whether they had difficulty uploading pictures of their food (strongly disagree=1, disagree, neither agree nor disagree, agree, or strongly agree=5), whether they had difficulty understanding the questions (strongly disagree=1, disagree, neither agree nor disagree, agree, or strongly agree=5), and whether they had difficulty entering their responses (strongly disagree=1, disagree, neither agree nor disagree, agree, or strongly agree=5). Participant acceptability questions ranged from strongly disagree (value of 1) to strongly agree (value of 5); therefore, in these questions, a higher score correlated with a higher degree of difficulty for that topic.

### Crowdsourced Labeling of Food Images

MTurk was used to process the images of participants’ meals and snacks. MTurk is a crowdsourcing distributed human intelligence platform that has been used to process images for biomedical research [30-32]. Users upload discrete human intelligence tasks (HITs) that Workers can complete quickly for a small payment. In this study, 1 HIT required assigning the number of a particular food item in an image. Workers receive feedback on their performance through user approval or rejection of the HITs. Users can also specify the Worker qualifications to improve the quality of their responses.

**Figure 1:**
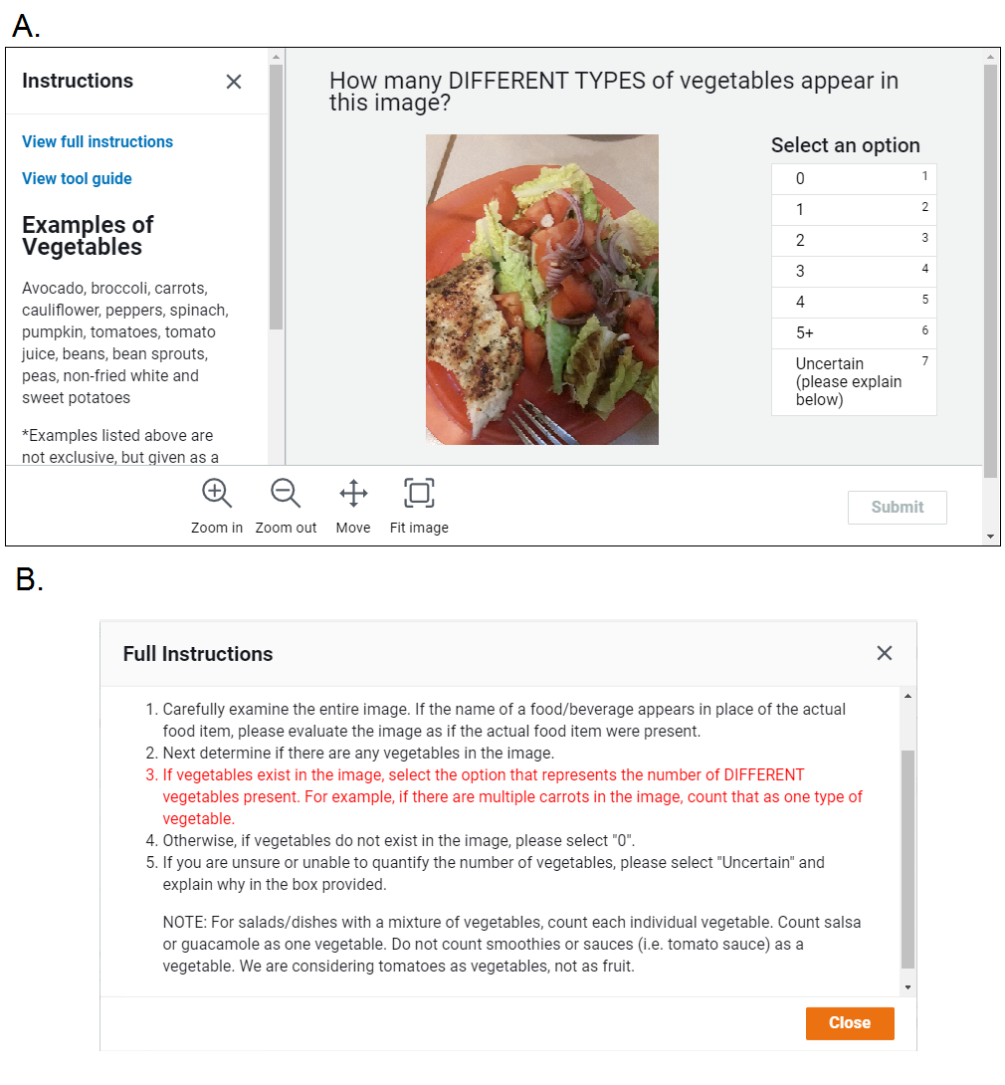
Example Mechanical Turk Worker interface for vegetable tasks, including an image with examples and the full instructions.

For this study, Workers were required to have >1000 approved HITs, with an approval rate of ≥99%, and they had to be located in the United States. Eligible Workers are randomly assigned to HITs and can complete as many as they choose, resulting in many Workers completing an assignment. In this study, Workers were asked to assign counts of the following food categories in separate HITs: fruits, vegetables, salty calorie-dense foods (eg, potato chips and fries), and sweet calorie-dense foods (eg, cake, cookies, ice cream, candy, chocolate, and other pastries). Workers only counted the different food items within the current category, and they were not asked to quantify by serving size. For example, if the category was fruit and the image contained 2 grapes and half an apple, the count assigned would be 2, although it may not be equivalent to 2 servings of fruit. This is consistent with the type of eating behavior data collected in other EMA studies [10,14,33]. Screenshots of the instructions provided to Workers can be found in Figure 1.

Before uploading the images, a study team member checked each image to ensure that they did not accidentally include the participants’ personal information or anything that could potentially identify participants. Each batch contained approximately 100 images (HITs), and although participants were given 1 hour to complete each HIT (ie, assign the number of food items in 1 image), on average, whole batches were completed in 1 hour and 45 minutes (SD 69 minutes). Batches can be run simultaneously; therefore, all images could be processed within the same 2-hour period. Each HIT was estimated to take the Workers 15 seconds to complete, and they were provided a US $0.05 reward upon completion of each HIT. This is equivalent to US $13 per hour, which was the minimum wage in the city of Chicago at the time of the study.

Figure 1 displays the interface that MTurk Workers were presented. Along with the prompt, “How many different types of [food category] appear in this image?”, the Workers were provided with examples of the food in question and detailed instructions. Workers could select *0*, *1*, *2*, *3*, *4*, *5+*, or *uncertain* to categorize the image (Figure 1). If *uncertain* was selected, the Worker was asked to elaborate in the space provided.

### Participant Image–Captured Food Frequency

Responses from the MTurk Workers were downloaded as comma-separated value files in the same batches as the images were uploaded. Files were combined and cleaned to ensure the absence of duplicates. As 2 Workers were assigned to count the food items for each image, their responses were compared. Images with discordant responses between Workers were evaluated by a study team member and given a final count. Weekly intake was calculated for each participant by summing the items across the images by participant ID for each food category (fruits, vegetables, salty snacks, and sweet snacks) using the final counts assigned to the images.

### Statistical Analysis

Acceptability of photographing food and uploading the images was evaluated based on responses to survey questions and the total number of photographs taken by participants over the EMA study period. Survey responses were categorized on Likert scales, and mean values were calculated for each component across participants. The feasibility of using MTurk to count the number of fruits, vegetables, sweet snacks, and salty snacks for each participant was assessed by calculating the percentage agreement between the responses provided by the 2 MTurk Workers. Qualitative analyses were performed to better understand the reasons for any discordance between Workers; specifically, the image was assessed for the likely reason behind the discordance, the reasons were grouped into common themes, and the frequency of each theme was calculated separately for each food category. Weekly mean food intake by category was calculated for the food images and the Screener responses. Mean values were compared using 2-tailed *t* tests.

## Results

### Demographics

Table 1 lists the study participants’ sociodemographic characteristics. The average age was 37.5 years. Of the 48 participants, 23 (48%) identified as non-Hispanic White, 5 (10%) as non-Hispanic Black, and 19 (40%) as Hispanic or Latina. Approximately 68% (32/48) of participants had at least a bachelor’s degree or higher, and 44% (21/48) found it somewhat hard or harder to pay for the basics. All participants were female.

**Table 1:** Participant characteristics (N=48).

Characteristics			Participants, n (%)
**Age (years)**			
	<40	30 (63)	
	≥40	18 (38)	
**Race^a^**			
	Non-Hispanic White	23 (48)	
	Non-Hispanic Black	5 (10)	
	Hispanic or Latina	19 (40)	
**Education^a^**			
	Less than a bachelor’s degree	15 (32)	
	Bachelor’s degree or higher	32 (68)	
**Financial burden^a^**			
	Not very hard to pay for basics	27 (56)	
	Hard to pay for basics	21 (44)	

^a^One participant was missing demographic information.

### Acceptability and Data Quality

A total of 1022 images were collected by the participants. Approximately 3.03% (31/1022) of images contained a written description of what was eaten in English, and 0.78% (8/1022) of images contained a description written in Spanish. Images containing a description in English were included in the batches uploaded to MTurk, and the counts were assigned by the Workers. Images with Spanish descriptions were translated, and a trained study team member assigned the counts of fruits, vegetables, salty snacks, and sweet snacks. These images were not uploaded to MTurk but were included in the final analyses comparing image and Screener responses.

Table 2 provides the average survey responses. Participants reported no difficulty in entering responses (mean 1.40, SD 0.71), understanding the questions (mean 1.48, SD 0.90), or uploading photographs (mean 2.15, SD 1.24). An average of 21.3 photographs (SD 9.52) per participant were taken over the study period. Participants remembered to take photographs more than half of the time on average, and their behavior changed slightly to somewhat because of participation in the study.

**Table 2:** Responses to survey questions on participant acceptability and data quality.

Measure			Score, mean (SD)
**Participant acceptability^a^**			
	Difficulty entering responses	1.40 (0.71)	
	Difficulty understanding questions	1.48 (0.90)	
	Difficulty uploading photographs	2.15 (1.24)	
**Data quality**			
	Number of photographs taken	21.29 (9.52)	
	Remembered to take photographs^b^	3.85 (0.87)	
	How much did taking pictures change behavior^c^	2.38 (1.18)	

^a^Possible options for each question in this section ranged from strongly disagree (1) to strongly agree (5).

^b^Possible options ranged from none of the time (1) to all of the time (5).

^c^Possible options ranged from not at all (1) to substantially (5).

### Feasibility of Using MTurk

After the 7-day study period, 99.22% (1014/1022) of photographs of participants’ meals and snacks were assessed by MTurk Workers. Each image was uploaded and evaluated for the presence of fruits, vegetables, salty snacks, and sweet snacks; therefore, 4056 HITs were completed by Workers. On average, the batches took 1 hour and 45 minutes to process. Classification agreement among the MTurk Workers was moderate for vegetables (688/1014, 67.85%; images received the same response from both Workers), and agreement was high for all other food categories (871/1014, 85.89% for fruits; 847/1014, 83.53% for salty snacks; and 833/1014, 82.15% for sweet snacks; images received the same response). The study team performed a thematic analysis of the images that received discordant responses. A total of 4 categories were identified that presented possible explanations for the discordance: poor image quality, image subject uncertainty, user error, and miscellaneous. Images in the poor image quality category were blurry, had low visibility or a dark contrast, or the background was confusing or misleading. The image subject uncertainty category included foods that may have been difficult to discern or that portrayed a mixture of several items, such as salads, rice bowls, or vegetable mixes. In the user error category, image answers were in contrast to the provided MTurk instructions (ie, Workers were instructed to count tomatoes as its nutritional designation, as a vegetable, despite botanically being classified as a fruit), or the Worker inaccurately counted the types of food in question. The miscellaneous category applied to images that failed to fit into these 3 main classes.

**Figure 2:**
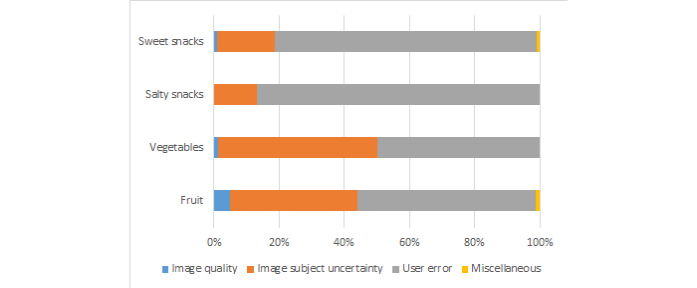
Prevalence of discordant Mechanical Turk Worker response explanations by food category.

**Figure 3:**
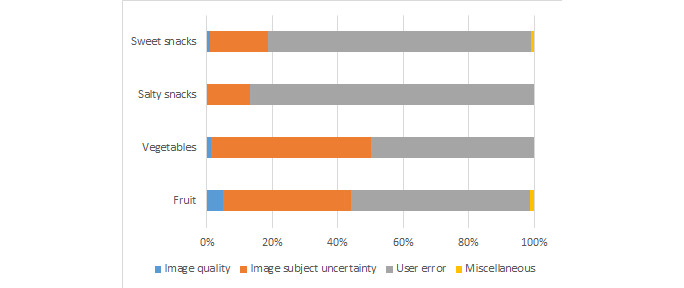
Prevalence of discordant Mechanical Turk Worker responses by types of user error.

Figure 2 presents the prevalence of the 4 types of discordant response explanations by food category. User error was the most prevalent reason for discordant responses across all food categories: it was most prevalent in salty snacks (145/167, 86.8% of images with discordant responses), followed by sweet snacks (145/181, 80.1%), fruits (78/143, 54.5%), and finally vegetables (163/326, 50%). Figure 3 further breaks down user errors into its subgroups: incorrect responses despite instruction clarification and incorrect responses because of Worker inaccuracies not specifically addressed in the instructions. Within these groups, the latter was most prevalent across all food types, with the highest percentage in salty snacks (135/145, 93.1% of images) and the lowest percentage in fruits (41/78, 53% of images).

Image subject uncertainty was the next most common explanation for discordant responses. This explained 48.8% (159/326) of vegetable discordances, 39.2% (56/143) of fruit discordances, 17.7% (32/181) of sweet snacks discordances, and 13.2% (22/167) of salty snacks discordances (Figure 2). Image quality explained 1.2% (4/326) of vegetables, 4.9% (7/143) of fruit, 0% (0/167) of salty snacks, and 1.1% (2/181) of sweet snacks discordances. Finally, only 0.39% (4/1014) of responses were grouped into the miscellaneous category: 50% (2/4) in the fruit and 50% (2/4) in the sweet snacks responses.

### Comparison of Usual Consumption From Food Images and Dietary Screener

The food images and the Screener were able to capture similar patterns of food intake. In both methods, vegetables were reportedly consumed most frequently and fruits least frequently (Table 3). Although the frequency of sweet snack consumption was lower using food images compared with that using the Screener, there were no statistically significant differences in the weekly frequency of food consumption between the image and the Screener results across all 4 food categories (fruit, *P*=.99; vegetable, *P*=.54; salty snacks, *P*=.56; and sweet snacks, *P*=.37).

**Table 3:** Weekly food consumption (times per week) based on food images and the Block Screener.

Food category		Frequency, mean (SD)
**Fruit**		
	Images	3.58 (4.20)
	Block Screener	3.59 (2.32)
**Vegetables**		
	Images	9.96 (6.01)
	Block Screener	10.28 (4.58)
**Salty snacks**		
	Images	4.17 (3.21)
	Block Screener	3.85 (3.16)
**Sweet snacks**		
	Images	3.77 (4.13)
	Block Screener	4.51 (4.13)

No statistically significant differences were found in food consumption levels between the MTurk-processed images and the Screener across all food categories.

## Discussion

### Principal Findings

This pilot study demonstrates the feasibility of collecting data on food intake through participant-captured and crowdsource-labeled images. The method of photographing eating events in the context of an EMA study was generally well-accepted and executed by the study participants. Most remembered to take pictures of their meals and snacks, and few reported difficulties in uploading the images. Importantly, uploading the photographs was not more difficult for participants than entering the responses. Images could be processed in a timely manner, and there was high agreement in the MTurk Worker count responses, particularly for fruits, salty snacks, and sweet snacks images, thereby supporting the feasibility of using MTurk for image classification. In addition, the weekly consumption estimated by the food images and the Screener was comparable.

Overall, vegetables were reportedly consumed more frequently compared with the other 3 food groups, and this was consistent between the 2 methods. Neither method aimed to measure the serving size but rather the frequency that these food groups were eaten. Both the MTurk instruction and the Screener aimed to measure the types of food eaten; therefore, in 1 meal or snack, multiple vegetables might have been present and been the driving force behind higher numbers.

This study further supports the use of participant-captured images to assess eating events. Compared with traditional EMA methods such as completing surveys or dietary journals, this study had comparable compliance rates: participants remembered to take photographs 77% (3.85/5) of the time [21,34,35]. Previous studies have used participant-captured food images and found high acceptability and data quality, with participant compliance rates ranging from 30% to 60% [16-18,36]. These studies had used dieticians to assess the energy intake or macronutrient levels from the images; therefore, our study demonstrates a novel approach of coupling participant-captured food images with crowdsourced image labeling. This method can accurately assess eating events in an EMA study without a time-intensive dietician review.

On average, batches of images took 1 hour and 45 minutes to process. Batches could be run simultaneously, allowing many images to be labeled in the same time frame. Theoretically, if a user could start all batches at the same time, all 4056 HITs could be accomplished within the same 1 hour and 45 minutes. The most limiting factor for time efficiency was the user’s ability to prepare and publish the batches of images. Even so, using MTurk to crowdsource image identification is more time-efficient compared with the participants or study team members labeling the images. This is especially significant for future studies that wish to scale up this model with more participants or expand food labeling outside of the 4 food options examined in this study.

Agreement in Worker responses using MTurk was high for most food categories; however, for vegetables, they only had a moderate agreement. Among the images that received discordant vegetable responses, approximately 50% were because of image subject uncertainty, the highest across all food categories. In this discordance explanation category, the subject of the image presented a meal or snack that made it difficult to discern the count for the type of food present. Typically, this involved a mixture of foods with some items hidden beneath others or a variety that was difficult to differentiate. Vegetable discordance may have been higher as these foods are more often eaten mixed within foods, such as salads, guacamole, rice bowls, and soups, compared with the other food groups. Future work is needed to optimize the accuracy of vegetable intake using MTurk.

Crowdsourcing with MTurk has been used successfully in several areas of biomedical research, including endoscopic video image annotation [32] and optic disc image classification [31]. Other studies have reported the feasibility of using MTurk for crowdsourcing nutritional analysis from images of meals [30]. This system, called PlateMate, estimated the macronutrient calories from fat, carbohydrates, and proteins shown, which is comparable with assessments among trained dietitians. Compared with end-of-day recall, crowdsourcing limits participant burden and self-reflection. Crowdsourcing allows many Workers to label images at the same time, cutting down the total time it would take participants or study team members to process the images. This action removes the obligation from study participants, lessening their burden. It also limits participant reactivity by reflecting on their eating choices, thus biasing the results when participants change their behaviors because of study procedures. Our pilot study supports the feasibility of using crowdsourcing to process images and offers the potential enhancement of EMA studies by using crowdsourcing to accurately capture eating events in real time.

### Limitations

The small sample size and limited socioeconomic diversity in our sample require replication in study populations with lower levels of education and income. The widespread availability of smartphones across socioeconomic groups supports our findings, despite the recognized digital divide [37]. This study used only female participants; therefore, further studies may benefit from recruiting both men and women to determine the feasibility of using smartphones and crowdsourcing to assess eating behaviors. Another limitation involved the inability to capture the daily frequency that participants ate a certain food item from the Screener; thus, the measure may not be directly analogous to the information captured with the food images. However, with the exception of fruits, multiple questions within the food categories were used to capture consumption, so our measures may more closely reflect instances rather than days.

Along the same lines, we were only able to evaluate and compare the number of times a participant ate a particular food with the food images, as opposed to assessing the serving size or the amount of the meal that was actually consumed. Most EMA studies are designed to assess eating behaviors on a micro timescale; thus, future studies would benefit from incorporating portion size as well. Seto et al [38] demonstrated the feasibility of using voice-annotated videos of meals and snacks to accurately capture portion sizes; however, trained dietitians were involved rather than crowdsourcing. The benefits of documenting portion size versus the time and costs of collecting and processing these data require further consideration depending upon the study aims.

### Conclusions

This pilot study demonstrates the feasibility of using participant-captured images categorized through a crowdsourcing platform to accurately depict eating events. This approach offers a potentially time-efficient and cost-effective strategy for EMA studies of this type. It can provide a richer breadth of data that reduces recall and reactivity biases in EMA studies compared with previous methods, such as dietary surveys and journals. It also offers an alternative strategy that is less burdensome to participants than previous EMA methods, as it reduces the amount of recall required by the participant. Our findings support the use of food images as a way of facilitating the growing interest in measuring food group frequency and general eating behaviors in a consumer-friendly manner with minimal additional burden.

## Abbreviations

CHEERS: Chicago Healthy Eating Environments and Resources Study
EMA: ecological momentary assessment
HIT: human intelligence task
MTurk: Mechanical Turk

## References

[1] Micha R, Kalantarian S, Wirojratana P, Byers T, Danaei G, Elmadfa I, Ding E, Giovannucci E, Powles J, Smith-Warner S, Ezzati M, Mozaffarian D, Global Burden of Diseases‚ NutritionChronic Disease Expert Group (2012). Estimating the global and regional burden of suboptimal nutrition on chronic disease: methods and inputs to the analysis. Eur J Clin Nutr.

[2] Micha R, Shulkin ML, Peñalvo JL, Khatibzadeh S, Singh GM, Rao M, Fahimi S, Powles J, Mozaffarian D (2017). Etiologic effects and optimal intakes of foods and nutrients for risk of cardiovascular diseases and diabetes: Systematic reviews and meta-analyses from the Nutrition and Chronic Diseases Expert Group (NutriCoDE). PLoS One.

[3] Willett WC, Stampfer MJ (2013). Current evidence on healthy eating. Annu Rev Public Health.

[4] Virani SS, Alonso A, Benjamin EJ, Bittencourt MS, Callaway CW, Carson AP, Chamberlain AM, Chang AR, Cheng S, Delling FN, Djousse L, Elkind MS, Ferguson JF, Fornage M, Khan SS, Kissela BM, Knutson KL, Kwan TW, Lackland DT, Lewis TT, Lichtman JH, Longenecker CT, Loop MS, Lutsey PL, Martin SS, Matsushita K, Moran AE, Mussolino ME, Perak AM, Rosamond WD, Roth GA, Sampson UK, Satou GM, Schroeder EB, Shah SH, Shay CM, Spartano NL, Stokes A, Tirschwell DL, VanWagner LB, Tsao CW, American Heart Association Council on Epidemiology and Prevention Statistics Committee and Stroke Statistics Subcommittee (2020). Heart disease and stroke statistics-2020 update: a report from the American Heart Association. Circulation.

[5] Afshin A, Sur PJ, Fay KA, Cornaby L, Ferrara G, Salama JS, Mullany EC, Abate KH, Abbafati C, Abebe Z, Afarideh M, Aggarwal A, Agrawal S, Akinyemiju T, Alahdab F, Bacha U, Bachman VF, Badali H, Badawi A, Bensenor IM, Bernabe E, Biadgilign SK, Biryukov SH, Cahill LE, Carrero JJ, Cercy KM, Dandona L, Dandona R, Dang AK, Degefa MG, Zaki ME, Esteghamati A, Esteghamati S, Fanzo J, Farinha CS, Farvid MS, Farzadfar F, Feigin VL, Fernandes JC, Flor LS, Foigt NA, Forouzanfar MH, Ganji M, Geleijnse JM, Gillum RF, Goulart AC, Grosso G, Guessous I, Hamidi S, Hankey GJ, Harikrishnan S, Hassen HY, Hay SI, Hoang CL, Horino M, Ikeda N, Islami F, Jackson MD, James SL, Johansson L, Jonas JB, Kasaeian A, Khader YS, Khalil IA, Khang Y, Kimokoti RW, Kokubo Y, Kumar GA, Lallukka T, Lopez AD, Lorkowski S, Lotufo PA, Lozano R, Malekzadeh R, März W, Meier T, Melaku YA, Mendoza W, Mensink GB, Micha R, Miller TR, Mirarefin M, Mohan V, Mokdad AH, Mozaffarian D, Nagel G, Naghavi M, Nguyen CT, Nixon MR, Ong KL, Pereira DM, Poustchi H, Qorbani M, Rai RK, Razo-García C, Rehm CD, Rivera JA, Rodríguez-Ramírez S, Roshandel G, Roth GA, Sanabria J, Sánchez-Pimienta TG, Sartorius B, Schmidhuber J, Schutte AE, Sepanlou SG, Shin M, Sorensen RJ, Springmann M, Szponar L, Thorne-Lyman AL, Thrift AG, Touvier M, Tran BX, Tyrovolas S, Ukwaja KN, Ullah I, Uthman OA, Vaezghasemi M, Vasankari TJ, Vollset SE, Vos T, Vu GT, Vu LG, Weiderpass E, Werdecker A, Wijeratne T, Willett WC, Wu JH, Xu G, Yonemoto N, Yu C, Murray CJ (2019). Health effects of dietary risks in 195 countries, 1990–2017: a systematic analysis for the Global Burden of Disease Study 2017. Lancet.

[6] Robinson E, Blissett J, Higgs S (2013). Social influences on eating: implications for nutritional interventions. Nutr Res Rev.

[7] Caspi CE, Sorensen G, Subramanian S, Kawachi I (2012). The local food environment and diet: a systematic review. Health Place.

[8] Hardcastle S, Thøgersen-Ntoumani C, Chatzisarantis N (2015). Food choice and nutrition: a social psychological perspective. Nutrients.

[9] Shepherd R (1999). Social determinants of food choice. Proc Nutr Soc.

[10] Dunton GF (2018). Sustaining health-protective behaviors such as physical activity and healthy eating. J Am Med Assoc.

[11] Shiffman S, Stone AA, Hufford MR (2008). Ecological momentary assessment. Annu Rev Clin Psychol.

[12] Elliston KG, Ferguson SG, Schüz B (2017). Personal and situational predictors of everyday snacking: an application of temporal self-regulation theory. Br J Health Psychol.

[13] Schüz B, Revell S, Hills AP, Schüz N, Ferguson SG (2017). Higher BMI is associated with stronger effects of social cues on everyday snacking behaviour. Appetite.

[14] Zenk SN, Horoi I, McDonald A, Corte C, Riley B, Odoms-Young AM (2014). Ecological momentary assessment of environmental and personal factors and snack food intake in African American women. Appetite.

[15] Maugeri A, Barchitta M (2019). A systematic review of ecological momentary assessment of diet: implications and perspectives for nutritional epidemiology. Nutrients.

[16] Schembre SM, Liao Y, O'Connor SG, Hingle MD, Shen S, Hamoy KG, Huh J, Dunton GF, Weiss R, Thomson CA, Boushey CJ (2018). Mobile ecological momentary diet assessment methods for behavioral research: systematic review. JMIR Mhealth Uhealth.

[17] Ashman A, Collins C, Brown L, Rae K, Rollo M (2017). Validation of a smartphone image-based dietary assessment method for pregnant women. Nutrients.

[18] Boushey C, Spoden M, Delp E, Zhu F, Bosch M, Ahmad Z, Shvetsov Y, DeLany J, Kerr D (2017). Reported energy intake accuracy compared to doubly labeled water and usability of the mobile food record among community dwelling adults. Nutrients.

[19] Berkman ET, Giuliani NR, Pruitt AK (2014). Comparison of text messaging and paper-and-pencil for ecological momentary assessment of food craving and intake. Appetite.

[20] Strahler J, Nater UM (2018). Differential effects of eating and drinking on wellbeing - an ecological ambulatory assessment study. Biol Psychol.

[21] Grenard JL, Stacy AW, Shiffman S, Baraldi AN, MacKinnon DP, Lockhart G, Kisbu-Sakarya Y, Boyle S, Beleva Y, Koprowski C, Ames SL, Reynolds KD (2013). Sweetened drink and snacking cues in adolescents: a study using ecological momentary assessment. Appetite.

[22] Ashurst J, van Woerden I, Dunton G, Todd M, Ohri-Vachaspati P, Swan P, Bruening M (2018). The association among emotions and food choices in first-year college students using mobile-ecological momentary assessments. BMC Public Health.

[23] Bejarano CM, Cushing CC (2018). Dietary motivation and hedonic hunger predict palatable food consumption: an intensive longitudinal study of adolescents. Ann Behav Med.

[24] Berge JM, Tate A, Trofholz A, Fertig A, Crow S, Neumark-Sztainer D, Miner M (2018). Examining within- and across-day relationships between transient and chronic stress and parent food-related parenting practices in a racially/ethnically diverse and immigrant population : Stress types and food-related parenting practices. Int J Behav Nutr Phys Act.

[25] Ram N, Brinberg M, Pincus AL, Conroy DE (2017). The questionable ecological validity of ecological momentary assessment: considerations for design and analysis. Res Hum Dev.

[26] Afshinnekoo E, Ahsanuddin S, Mason CE (2016). Globalizing and crowdsourcing biomedical research. Br Med Bull.

[27] Kershaw KN, Klikuszowian E, Schrader L, Siddique J, Van Horn L, Womack VY, Zenk SN (2019). Assessment of the influence of food attributes on meal choice selection by socioeconomic status and race/ethnicity among women living in Chicago, USA: a discrete choice experiment. Appetite.

[28] Doherty TJ (2001). The influence of aging and sex on skeletal muscle mass and strength. Curr Opin Clin Nutr Metab Care.

[29] Lalonde I, Graham M, Slovinec-D'Angelo M, Beaton L, Brown J, Block T (2008). Validation of the block fat/sugar/fruit/vegetable Screener in a cardiac rehabilitation setting. J Cardiopulm Rehabil Prev.

[30] Noronha J, Hysen E, Zhang H, Gajos KZ (2011). Platemate: crowdsourcing nutritional analysis from food photographs. Proceedings of the 24th Annual Acm Symposium on User Interface Software and Technology.

[31] Brady CJ, Villanti AC, Pearson JL, Kirchner TR, Gupta OP, Shah CP (2014). Rapid grading of fundus photographs for diabetic retinopathy using crowdsourcing. J Med Internet Res.

[32] Maier-Hein L, Mersmann S, Kondermann D, Stock C, Kenngott HG, Sanchez A, Wagner M, Preukschas A, Wekerle A, Helfert S, Bodenstedt S, Speidel S (2014). Crowdsourcing for reference correspondence generation in endoscopic images. Proceedings of the International Conference on Medical Image Computing and Computer-Assisted Intervention.

[33] Liao Y, Skelton K, Dunton G, Bruening M (2016). A systematic review of methods and procedures used in ecological momentary assessments of diet and physical activity research in youth: an adapted STROBE checklist for reporting EMA studies (CREMAS). J Med Internet Res.

[34] Thomas JG, Doshi S, Crosby RD, Lowe MR (2011). Ecological momentary assessment of obesogenic eating behavior: combining person-specific and environmental predictors. Obesity (Silver Spring).

[35] Ghosh Roy P, Jones KK, Martyn-Nemeth P, Zenk SN (2019). Contextual correlates of energy-dense snack food and sweetened beverage intake across the day in African American women: an application of ecological momentary assessment. Appetite.

[36] Six BL, Schap TE, Zhu FM, Mariappan A, Bosch M, Delp EJ, Ebert DS, Kerr DA, Boushey CJ (2010). Evidence-based development of a mobile telephone food record. J Am Diet Assoc.

[37] Vogels EA (2021). Digital divide persists even as Americans with lower incomes make gains in tech adoption. Pew Research Center.

[38] Seto E, Hua J, Wu L, Shia V, Eom S, Wang M, Li Y (2016). Models of individual dietary behavior based on smartphone data: the influence of routine, physical activity, emotion, and food environment. PLoS One.

